# Parasitic infections among pregnant women at first antenatal care visit in northern Ghana: A study of prevalence and associated factors

**DOI:** 10.1371/journal.pone.0236514

**Published:** 2020-07-24

**Authors:** Benjamin Ahenkorah, Kwabena Nsiah, Peter Baffoe, Winfred Ofosu, Charles Gyasi, Eddie-Williams Owiredu

**Affiliations:** 1 Department of Molecular Medicine, School of Medicine and Dentistry, Kwame Nkrumah University of Science and Technology, Kumasi, Ghana; 2 Biochemistry and Hematology Units, Bolgatanga Regional Hospital, Bolgatanga-Upper East Region, Ghana; 3 Department of Biochemistry and Molecular Medicine, School of Medical and Health Science, University for Development Studies, Tamale, Ghana; 4 Department of Biochemistry and Biotechnology, Kwame Nkrumah University of Science and Technology, Kumasi, Ghana; 5 United Nations Children’s Fund, Accra, Ghana; 6 Ghana Health Service, Accra, Ghana; 7 Department of Medical Diagnostics, Faculty of Allied Health Sciences, Kwame Nkrumah University of Science and Technology, Kumasi, Ghana; University of Virginia, UNITED STATES

## Abstract

**Background:**

Parasitic infections remain widespread in developing countries and constitute a major public health problem in many parts of sub-Saharan Africa. It is prevalent among children under 5 years and pregnant women; however, studies among the later high risk group is limited in the northern part of Ghana. Here, we evaluated the prevalence and associated factors of parasitic infections among pregnant women at first antenatal care visit in northern Ghana.

**Methods:**

This was a cross-sectional study conducted at the Department of Obstetrics and Gynecology, Bolgatanga Regional Hospital, Upper East Region-Ghana. A total of 334 consecutive consenting pregnant women were included. Questionnaires were administered to obtain socio-demographic data. Venous blood, stool and urine samples were collected for parasite identification using microscopy. Factors associated with parasitic infections were evaluated using regression models. Statistical analysis was performed using R.

**Results:**

Parasitic infections identified were giardiasis (30.5%), *P*. *falciparum* malaria (21.6%) and schistosomiasis (0.6%). Polyparasitic infection was identified in 6.6% of the population. Increasing age [Age of 20–29 years: AOR = 0.16, 95% CI (0.06–0.38); Age of 30–39 years: AOR = 0.21, 95% CI (0.08–0.50); Age >39 years: AOR = 0.30, 95% CI (0.11–0.83)] was associated with lower odds whiles presence of domestic animals [AOR = 1.85, 95% CI (1.01–3.39)], being in the second trimester of pregnancy [AOR = 2.21, 95% CI (1.17–4.19)], having no formal education [AOR = 3.29, 95% CI (1.47–7.35)] and basic education as the highest educational level [AOR = 6.03, 95% CI (2.46–10.81)] were independent predictors of increased odds of giardiasis. Similarly, having no formal education [AOR = 2.88, 95% CI (1.21–8.79)] was independently associated with higher odds of *P*. *falciparum* malaria. The use of insecticide treated net (ITN) [AOR = 0.43, 95% CI (0.21–0.89)] and mosquito repellent [AOR = 0.09, 95% CI (0.04–0.21)] were independent predictors of lower odds of *P*. *falciparum* malaria.

**Conclusion:**

Giardiasis and *P*. *falciparum* malaria are common among pregnant women in northern Ghana. The major associated factors of giardiasis are lack of or low level of formal education, the presence of domestic animals and being in the second trimester of pregnancy. Increasing age confers protection against giardiasis. Likewise, lack of formal education is an associated factor for *P*. *falciparum* malaria among pregnant women in northern Ghana. The use of ITN and mosquito repellents reduce the risk of *P*. *falciparum* malaria. Given the possible role of parasitic infections in adverse pregnancy outcomes, our findings highlight the need for regular screening and treatment of infected women in the northern parts of Ghana. Public health education and improving socio-economic status could help reduce the risk of parasitic infections among pregnant women in the region.

## Introduction

Parasitic infections are a considered global public health problem. These infections are more prevalent in developing countries. The predominant parasitic infections in Africa are intestinal parasitic infections (IPIs) and malaria [1, 2].

IPIs are conditions in which a parasite infects the gastrointestinal tract of humans. A recent report by the World Health Organization (WHO) indicates that approximately 1.5 billion people, accounting for about 24% of the global population, are infected with intestinal parasites [1]. Although the rate of infection is declining in developing countries, the prevalence of IPIs remains high in sub Saharan Africa, particularly in settings with poor sanitary conditions [1].

IPIs are caused by either helminths, protozoa or both. In sub Saharan Africa, the prevalence of IPIs could reach up to 95% in some settings [3]. These infections have been linked with poor sanitation, lack of access to safe drinking water and poor hygiene. For this reason, some IPIs are considered diseases of poverty. During pregnancy, cell-mediated immunity is transiently depressed [4], interfering with the resistance mechanisms to various infectious diseases; thus, expectant mothers become more vulnerable to infections. Pregnant women with IPIs are at increased risk of maternal complications and adverse perinatal outcomes such as anemia, low birth weight and perinatal mortality [5]. Nonetheless, the effect of parasitic infections to a mother or an infant depends on the mother’s natural immunity, type of infecting parasite and parasitic load [6]. To reduce and eventually eliminate IPI-related morbidity, the WHO recommends that endemic countries practice periodic treatment (deworming) of at-risk people such as preschool and school-age children, women of reproductive age and adults in certain high-risk occupations [1].

Like IPIs, malaria is also prevalent in developing countries. Currently, the WHO estimates 219 million cases and 435,000 malaria-related deaths globally [2]. In the WHO African Region, malaria causes significant morbidity and mortality with annual infection and mortality rates of 213 million and 380,000 individuals, respectively [2]. In Ghana, malaria remains a major cause of loss of days of healthy life, accounting for not less than 20% of child deaths, 40% of child hospital admissions, and more than 50% of outpatient attendances [7, 8]. Globally, five species of *Plasmodium* (*falciparum*, *malariae*, *ovale*, *vivax and knowlesi*) are known to cause human malaria but *P*. *falciparum* is the predominant cause of the disease in Africa and Ghana, resulting in debilitating symptoms and complications and accounts for about 86% of all infections [9]. Similar to IPIs, both children, particularly those under 5 years old and pregnant women are at increased risk of malaria and its associated morbidity. When acquired during pregnancy, malaria can lead to anaemia, maternal death, spontaneous abortions, low birth weight, and neonatal death [10]. The enormous toll on life and both national and household economics [11] underscores the need for early malaria diagnosis, treatment and disease surveillance. In order to abate the risk of infection and ameliorate the effect of malaria in pregnancy, the WHO recommends the use of long-lasting insecticidal net and the administration of Sulphadoxine-Pyrimethamine during antenatal care (ANC) visits [8].

Although a number of studies on parasitic infections among pregnant women have been conducted across Ghana [12–16] and in neighboring African countries [17–20], there remains a dearth of published data among pregnant women in the northern parts of the Ghana where adequate health facilities are limited. Here, we evaluated the prevalence and associated factors of parasitic infections among pregnant women at first antenatal care visit in northern Ghana.

## Material and methods

### Study design/setting

This cross-sectional study was conducted at the Department of Obstetrics and Gynecology, Bolgatanga Regional Hospital, Upper East Region-Ghana, from May 2013 to May 2014. The Bolgatanga Regional Hospital is a referral hospital which provides primary healthcare and specialist referral services for the people in the northern sector of Ghana. It also serves as a training center for medical students, nurses, midwives, laboratory scientists, dentists, radiographers and pharmacists. In 2011, the antenatal coverage for the Bolgatanga Regional Hospital was almost 20% of the municipality’s expected pregnancies.

### Participants recruitment

**Fig 1:**
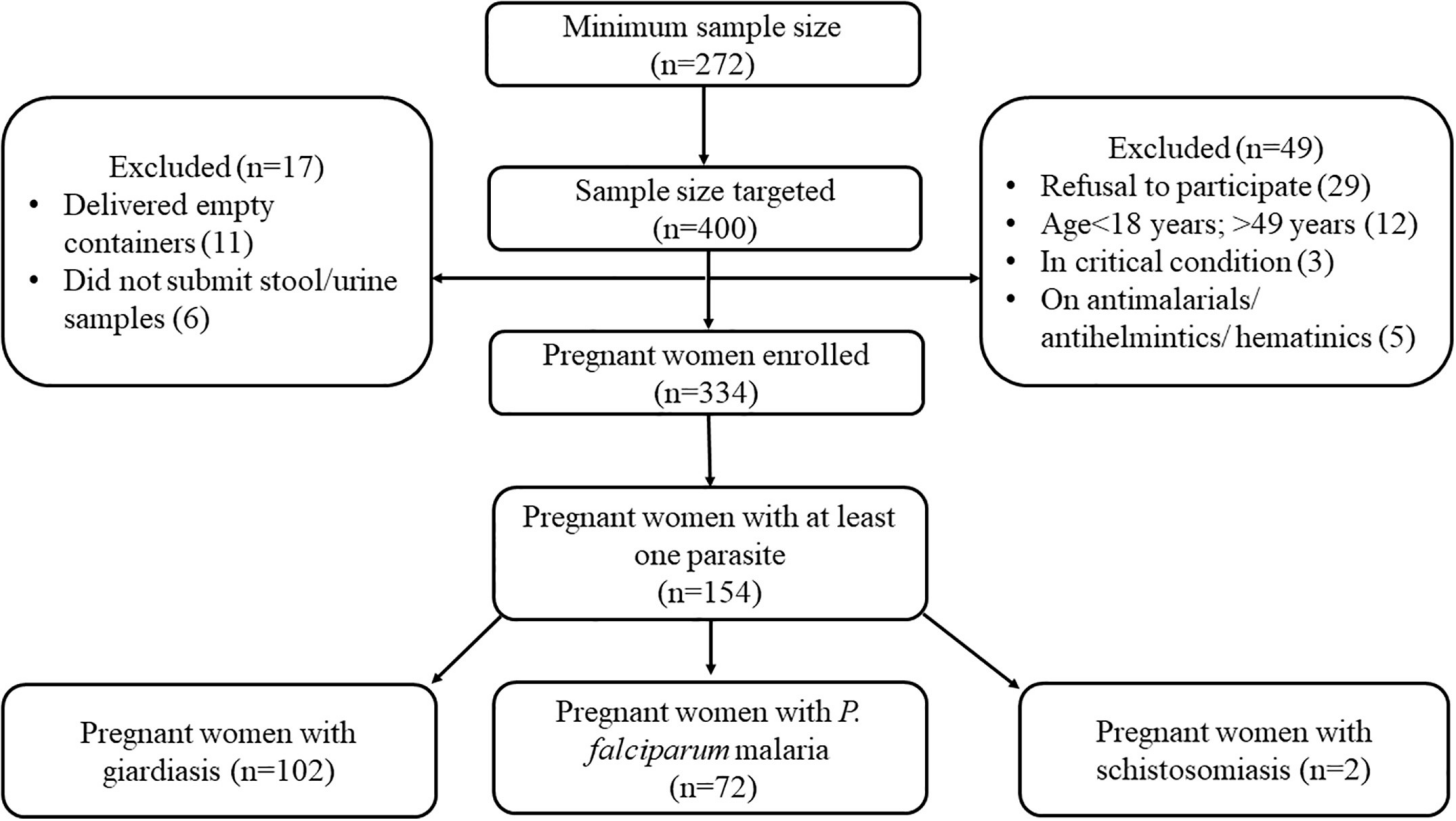
Flowchart of participation in the study.

The sample size for this study was calculated using the MedCalc Statistical Software version 18.9.1 (MedCalc Software bvba, Ostend, Belgium). Based on the most recent estimated prevalence of parasitic infections among pregnant women in northern Ghana (23%) [21], 95% confidence level, 5% margin of error, a study power of 80%, and design effect of 1, the minimum sample size required for this study was 272. However, in an effort to strengthen statistical power, we targeted a total of 400 participants for the study. Of this, 334 consecutive consenting pregnant women were included in the study. Excluded participants were pregnant women who were in critical condition and needed emergency care, those below 18 and above 49 years of age and those on antimalarials/ anthelmintics/ haematinics. All pregnant women who met the inclusion criteria and consented after the aim and objectives had been explained to them were eligible to participate in the study. The flowchart for participants’ selection is shown in Fig 1.

### Ethics approval and consent to participate

Ethical approval for this study was obtained from the Committee on Human Research, Publication and Ethics (CHRPE) of the School of Medical Sciences, Kwame Nkrumah University of Science and Technology (CHRPE/AP/104/13) and the institutional review board of the Navrongo Health Research Centre (NHRCIRB151). Written informed consent was obtained from all participants who opted to participate after the aims and objectives of the study had been explained to them. Participation was voluntary, and respondents were assured that the information obtained was strictly for research and academic purposes only and were guaranteed the liberty to opt out from the study at their own convenience.

### Questionnaire administration

Questionnaires, designed by reviewing studies of similar objective and tailored to fit our study objectives, were administered to obtain socio-demographic data from the participants. Data collected include age, gestational age, parity, highest educational level, residence, employment status, major source of food and drinking water, presence of domestic animals, use of mosquito repellent and insecticide treated net (ITN).

### Sample collection and assay

Five milliliters (5 ml) of venous blood was obtained under aseptic conditions and dispensed into K_3_ EDTA tubes. Haemoglobin level estimation was performed using the Sysmex KX-21 N Automated Hematology Analyzer (Sysmex Corporation Kobe, Japan); sickle cell screening and phenotyping were done using the 2% sodium metabisulphite technique and electrophoresis at alkaline pH, respectively. For malaria parasite screening by microscopy, ten percent Giemsa-stained thick and thin films were prepared on clean grease-free slides (thin films were fixed with methanol) [22]. Examination and reporting of both thick and thin films were performed independently by two trained microscopists. The tests were performed immediately after sample collection to minimize storage variabilities.

Additionally, to each participant, two well–labelled, wide-mouth and screw-capped containers were given and were instructed to bring their early morning stool and urine samples the following day. The stool and urine samples were immediately transported in cold boxes at 4°C to the Laboratory Department of the Bolgatanga Regional Hospital for processing and examination. In an effort to increase the detection rates, all the stool samples were initially screened by direct smear technique, followed by the formol-ether concentration method. Further, about 10 g of the stool was thoroughly mixed and fixed in polyvinyl alcohol for the detection of intestinal protozoa, using trichrome staining technique [23]. The urine sedimentation technique was used for the detection of *Schistosoma hematobium* ova. For all stool and urine parasitological tests, samples were considered as positive if the eggs/ova/cysts/trophozoites were detected. For quality control purposes, all slides were prepared and examined in duplicates.

## Data analysis

Statistical analysis was performed using the R Language for Statistical Computing version 3.6.0 [24]. Continuous data were presented as mean ± standard deviation (SD). Categorical data were presented as frequencies (percentages). To determine potential factors associated with the presence of a condition (giardiasis and malaria), we first performed univariate logistic regression analysis. This was followed by multivariate logistic regression analysis, using the enter method for variables with p-values < 0.05 after univariate analysis, to identify independent factors associated with the condition. We do not report regression analysis for schistosomiasis because the relatively low number of positive cases limited statistical reliability. All tests were two-sided and p-value < 0.05 was considered statistically significant.

## Results

### Characteristics of the population

A total of 334 pregnant women with mean age of 28.95 (SD: 6.14) years old were included in this study. Most of the participants had no formal education, resided in rural areas and were employed. More than half of the participants were multiparous, were in their second trimester of the current pregnancy and used ITN. The average hemoglobin level was 10.13 g/dL (Table 1).

**Table 1:** Baseline characteristics of the study population.

Variables	Mean ±SD	
**Age (years)**	28.95±6.14	
**Age ranges (years)**	**Frequency (n = 334)**	**Percentage (%)**
<30	170	50.9
>30	164	49.1
**Educational level**		
None	158	47.2
Basic/Secondary	108	32.3
Tertiary	68	20.4
**Residence**		
Urban	121	36.2
Rural	213	63.8
**Employment status**		
Employed	242	72.5
Unemployed	92	27.5
**Parity**		
Nulliparous	131	39.2
Multiparous	203	60.8
**Gestational age**		
First trimester	110	32.9
Second trimester	179	53.6
Third trimester	45	13.5
**Source of drinking water**		
Tap	130	38.9
Well	204	61.1
Borehole		
**Predominant source of food**		
Home-cooked	143	42.8
Street-bought	191	57.2
**Presence of livestock at home**		
No	133	39.8
Yes	201	60.2
ITN use*		
No	120	46.5
Yes	138	53.5
Use of mosquito repellent**		
No	151	53.7
Yes	130	46.3
**Hemoglobin genotype**		
AA	246	73.7
AC	36	10.8
AS	34	10.2
CC	4	1.2
SC	12	3.6
SS	2	0.6
	**Mean ±SD**	
Hemoglobin (g/dL)	10.13±1.42	

*: Total number of responders (258).

**: Total number of responders (281).

### Prevalence of parasitic infections

**Fig 2:**
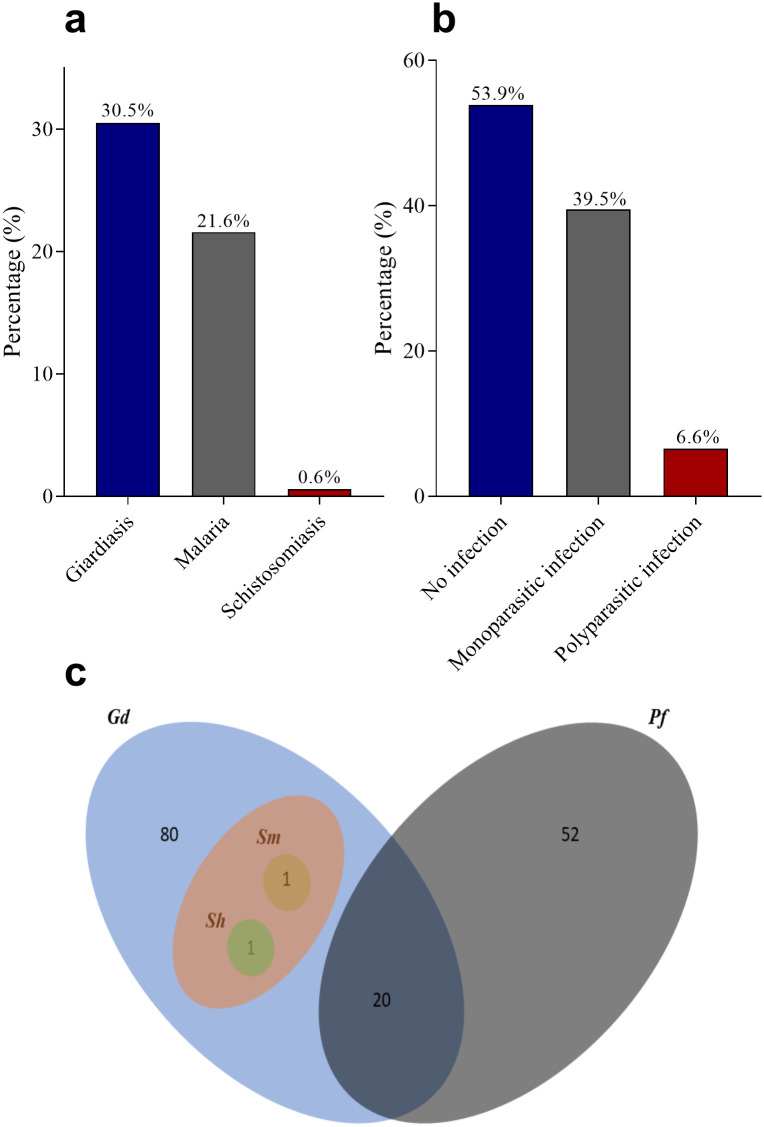
Prevalence of parasitic infections. *Gd*: *Giardia duodenalis*; *Sh*: *Schistosoma haematobium*; *Sm*: *Schistosoma mansoni*; *Pf*: *Plasmodium falciparum*. The figures in 2c represent the frequencies of occurrence of parasites.

Parasites infections identified in this study were giardiasis [30.5% (95% CI: 24.9–37.1)], malaria [21.6% (95% CI: 16.9–27.2)] and schistosomiasis [0.6% (95% CI: 0.1–2.3)] (Fig 2a) Only *P*. *falciparum* was detected. Among the participants, 39.5% (95% CI: 33.1–46.9) had monoparasitic infection comprising 80 and 52 were *Giardia duodenalis* and *Plasmodium falciparum*, respectively whereas 6.6% (95% CI: 4.1–9.9) had polyparasitic infection which consists of 2 *Giardia duodenalis*/Schistosomiasis co-infection (one *Schistosoma haematobium* and one *Schistosoma mansoni*) and 20 *Giardia duodenalis*/*Plasmodium falciparum* co-infections, respectively. There was no trio infection and helminthic infection was not identified (Fig 2b and 2c).

### Crude and adjusted odds ratios for factors potentially associated with giardiasis

**Fig 3:**
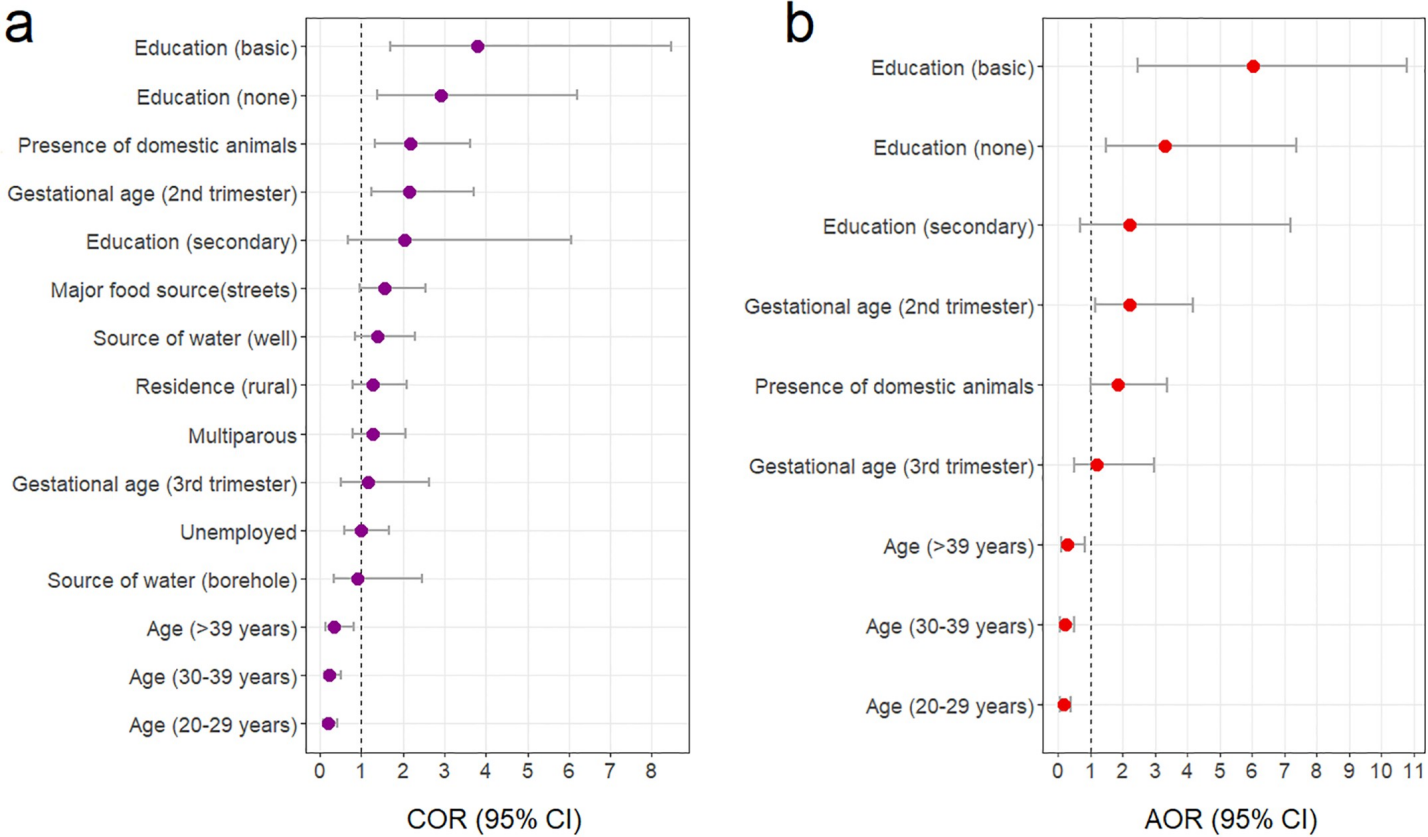
Crude and adjusted odds ratios for factors potentially associated with giardiasis. (**a**) Crude odds ratios; (**b**) adjusted odds ratios.

Using univariate logistic regression analysis, increasing age [Age of 20–29 years: COR = 0.19, 95% CI (0.08–0.43), p<0.0001; Age of 30–39 years: COR = 0.23, 95% CI (0.10–0.52), p<0.0001; Age >39 years: COR = 0.33, 95% CI (0.13–0.82), p = 0.017] was associated with lower odds of giardiasis whereas being in the second trimester of pregnancy at first ANC visit [COR = 2.14, 95% CI (1.24–3.70), p = 0.006], presence of domestic animals [COR = 2.19, 95% CI (2.19–3.62), p = 0.002], having no formal education [COR = 2.93, 95% CI (1.39–6.19), p = 0.005] and basic education as the highest educational level [COR = 3.79, 95% CI (1.69–8.48), p = 0.001] were associated with increased odds of giardiasis (Fig 3a). After adjusting for multiple covariates in a multivariate logistic regression analysis, increasing age [Age of 20–29 years: AOR = 0.16, 95% CI (0.06–0.38), p<0.0001; Age of 30–39 years: AOR = 0.21, 95% CI (0.08–0.50), p = 0.001; Age >39 years: AOR = 0.30, 95% CI (0.11–0.83), p = 0.020] remained associated with lower odds whiles presence of domestic animals [AOR = 1.85, 95% CI (1.01–3.39), p = 0.048], being in the second trimester of pregnancy at first ANC visit [AOR = 2.21, 95% CI (1.17–4.19), p = 0.015], having no formal education [AOR = 3.29, 95% CI (1.47–7.35), p = 0.004] and basic education [AOR = 6.03, 95% CI (2.46–10.81), p = 0.001] were independent predictors of increased odds of giardiasis (Fig 3b).

### Crude and adjusted odds ratios for factors potentially associated with P. falciparum malaria

**Fig 4:**
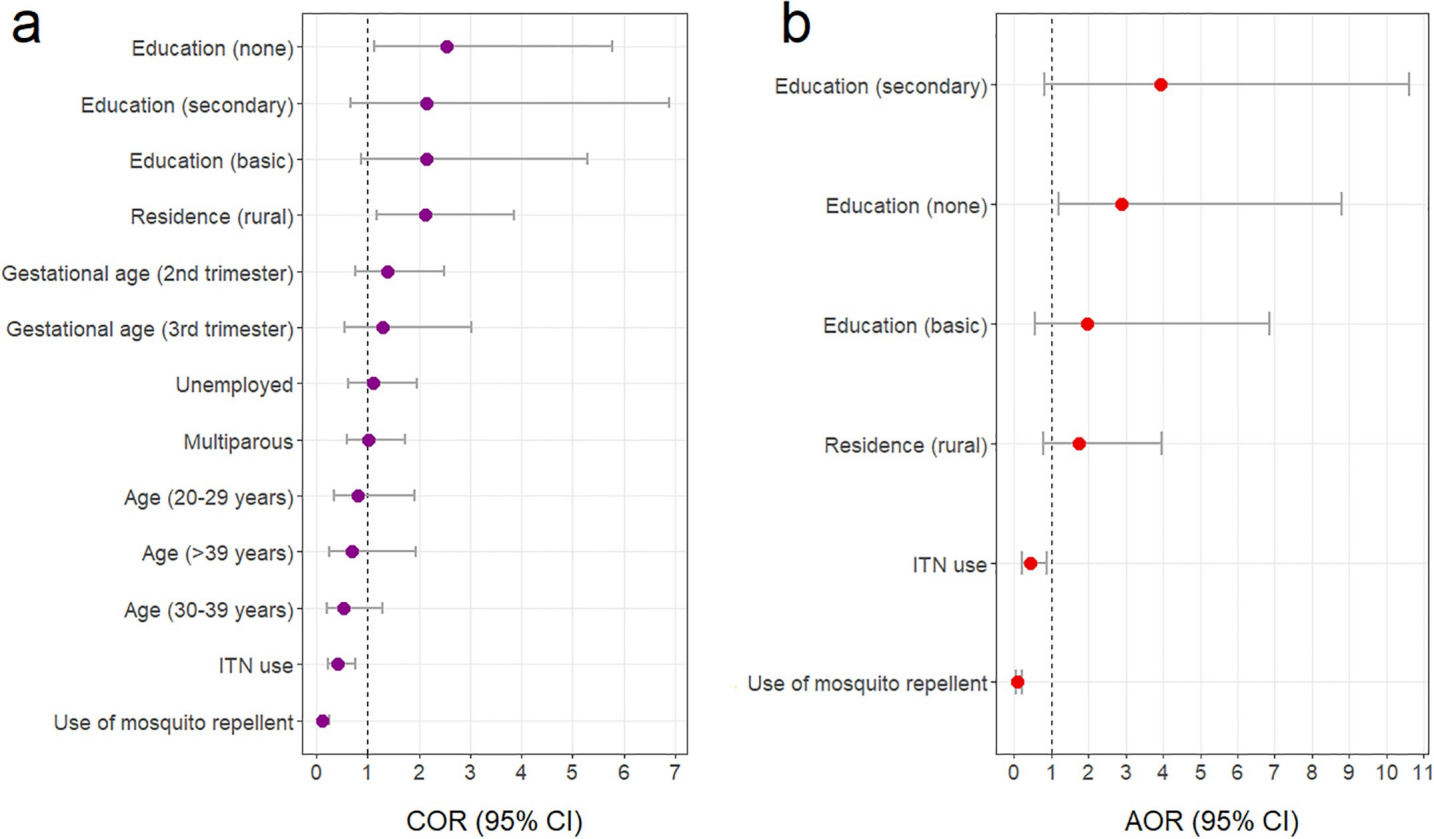
Crude and adjusted odds ratios for factors potentially associated with malaria. (**a**) Crude odds ratios; (**b**) adjusted odds ratios.

Univariate logistic regression analysis identified the use of mosquito repellent [COR = 0.12, 95% CI (0.06–0.25), p<0.0001] and ITN [COR = 0.40, 95% CI (0.22–0.76), p = 0.005] to be associated with lower odds whereas residing in rural areas [COR = 2.13, 95% CI (1.17–3.87), p = 0.013] and no formal education [COR = 2.54, 95% CI (1.12–5.77), p = 0.026] were associated with increased odds of *P*. *falciparum* malaria (Fig 4a). Adjusting for covariates in multivariate logistic regression analysis resulted in no formal education [AOR = 2.88, 95% CI (1.21–8.79), p = 0.033] being independently associated with higher odds whiles the use of ITN [AOR = 0.43, 95% CI (0.21–0.89), p = 0.023] and mosquito repellent [AOR = 0.09, 95% CI (0.04–0.21), p<0.0001] were independently associated with lower odds *P*. *falciparum* malaria (Fig 4b).

## Discussion

### Prevalence and associated factors of intestinal parasitic infections

Several studies on IPIs have been conducted in Ghana; however, these studies are predominantly among the pediatric population. Although a number of studies on IPIs have been conducted among pregnant women, the northern parts of the country is often overlooked. IPIs are caused by either helminths, protozoa or both; however, in this study among pregnant women from northern Ghana, we did not detect helminth infection. This could be due to the periodic national mass drug administration for helminth infestations and bilharzia conducted by the Ghana Health Service in an effort to reduce the burden of neglected tropical diseases in the country. It is thus not surprising that the prevalence of schistosomiasis was also very low (0.3% for *Schistosoma mansoni*). This notwithstanding, the prevalence of giardiasis was 30.5%. A much higher prevalence of 80% was reported by an earlier study in Accra-Ghana [12]. In other countries, varying prevalence rates of giardiasis have been reported among pregnant women. Gedefaw et al. [17] and Derso et al. [18] found prevalence of 18.84% and 13.3%, respectively, among pregnant women in Ethiopia. Rodriguez-Garcia et al. [25] and Espinosa et al. [26] reported a prevalence of 65.8% and 0.9% among pregnant women in Mexico and Colombia, respectively. The variations in the prevalence rates could be due to several factors including differences in geography, socio-economic conditions, cultural practices of the population and test methods used. Globally, the incidence of giardiasis is estimated at 280 million cases per year [27]. In developing countries, the prevalence ranges from 10 to 50% [28, 29]. Our finding thus falls within the commonly reported prevalence of giardiasis among the general population in developing countries. Of note, treatment of parasitic intestinal infections in pregnancy is complicated as some of the therapeutic interventions are considered unsafe during pregnancy; this could be the reason why testing among pregnant women is limited.

We do not report factors associated with schistosomiasis because of the limited number of positive cases. However, among the factors potentially associated with giardiasis, age, gestational age and educational level were identified as independent factors. Increasing age was associated with over 80% reduction in the likelihood of giardiasis compared to pregnant women with lower age. This finding could be related to the buildup of immunity to the infection with time [30]. On the other hand, pregnant women with domestic animals had about 2-fold increase in the odds of giardiasis. The infective stages of intestinal parasites are easily transmitted to humans by ingestion of the eggs/cyst/oocyst from contaminated sources [31]. In Ghana, particularly in the northern parts of the country, the extensive system of livestock production, where animals (predominantly cattle, goats and sheep) are kept free-range for part or all of their production cycle, is largely practiced [32]. Thus, open defecation by animals is common. Moreover, the people utilize the fecal materials of these animals as fertilizers. Together, these are practices that facilitate the contamination of the environment and thus contribute to the source of infection and spreading. Furthermore, women in their second trimester of pregnancy had increased odds of infection. This finding is consistent with a study by Derso et al. [18] who found that the odds of intestinal parasitic infection were increased by 22% in pregnant women in their second trimester. Other studies have also reported increased prevalence of intestinal parasite infections in the second trimester of pregnancy [31, 33]. Additionally, having no formal education or basic education as the highest educational level was identified as a predisposing factor for giardiasis. Consistent with our findings, higher educational level was identified to play a key role in the prevention of parasitic infections among pregnant women in Kenya [5] and Colombia [26]. These findings reinforce the concept that improving socio-economic status [34], particularly through educating people in endemic areas, would provide enormous help in reducing the prevalence of intestinal parasite infections among pregnant women.

### Prevalence and associated factors of *P*. *falciparum* malaria

The prevalence of *P*. *falciparum* malaria was 21.6%. Earlier studies in the region reported higher prevalence rates. A study by Fuseini et al. [35] observed a 58% prevalence of malaria among pregnant women in the Kassena‐Nankana district of Ghana. Another study by Clerk et al. [36] reported a 47% overall prevalence of malaria parasitaemia among pregnant women in northern Ghana. The lower prevalence in this study compared to early reports in the region highlights the effectiveness of the implementation of the vector management strategies through free distribution of insecticide-treated bed nets (ITN) in northern Ghana. Nonetheless, the prevalence of malaria in this study was higher than that of the national figure of ~18% [37]. Apart from the fact that pregnant women are a subset of the population, this could be as a result of the low patronage of the ITN by the people of middle and northern Ghana [38, 39]. Despite the limited number of studies in northern Ghana, a substantial number has been conducted in the southern parts of the country. In the Greater Accra region of Ghana, Stephens et al. [14] and Ofori et al. [13] reported 5.0% and 19.7% prevalence of *P*. *falciparum* parasitemia among pregnant women, respectively. In the Ashanti region of Ghana, Tay et al. [40] and Darko et al. [15] found a 12.6% and 19.0% prevalence of malaria among pregnant women. These differences in prevalence rates is an indication of intra- and inter-regional variations in *P*. *falciparum* infection rates and underscore the need for continual malaria surveillance, particularly in the northern parts of the country where adequate health facilities are wanting and the prevalence of malaria remains consistently higher than the other regions.

In assessing the factors associated with malaria in pregnancy, we found that having no formal education increases the likelihood of *P*. *falciparum* infection by approximately three-folds. Similar observation has been made by previous studies. Clerk et al. [36], in a study in northern Ghana also found that pregnant women with some level of education had about 30% reduction in the risk of malaria. Ndibazza et a. [19] and Woodburn et al. [20] also found education to be associated with reduced risk of malaria among pregnant women in Uganda. This finding may be explained by the connection between education, knowledge and disease prevention. Indeed, a study by Spjeldnæs et al. in Tanzania found that people who are educated had higher level of knowledge of malaria and over 2-fold increased likelihood of taking preventive actions against the infection [41]. As expected, the use of ITN and mosquito repellents reduced the risk of malaria in pregnancy by 57% and 99%, respectively. Indoor residual spraying with insecticides (IRS) and use of ITNs are some of the powerful tools recommended by the WHO to rapidly reduce malaria transmission in the presence of high level of coverage [42]. However, compliance to the use of ITN in the northern parts of Ghana remain consistently low, despite free periodic distribution of ITNs [38, 39]. Given that most of the pregnant women are uneducated, it is possible that these women lacked the understanding of the significance of ITNs and IRS use. We thus recommend that, in addition to strengthening the continuous and consistent distribution of ITNs, there is the need to fortify public health education on the effective preventive strategies of malaria as well as the importance of practicing them.

## Limitations of the study

This study is limited by the fact that the prevalence of intestinal parasitic infection was based on a single fecal sample instead of the ideal three consecutive samples. Therefore, the prevalence rate is likely to be underestimated. Additionally, storage of fecal samples in cold boxes at 4°C may have interfered with the parasitological diagnosis of strongyloidiasis and may account for the lack of positive cases. An on-site examination could help circumvent this limitation. Furthermore, despite the usefulness of microscopy in detecting parasitic infections, the low sensitivity of the method may have led to substantial number of missed cases, particularly in the presence of low intensity infections. Moreover, although the study was conducted at a referral center which serves most people from the northern part of Ghana, the findings of this study may not be generalizable to other areas. Further studies may also include nutritional assessment and density of infections which were not unavailable in this study.

## Conclusions

Giardiasis and *P*. *falciparum* malaria are common among pregnant women in northern Ghana. The major associated factor for giardiasis in northern Ghana are lack of and low level of formal education, the presence of domestic animals and being in the second trimester of pregnancy. Increasing age seems to confer protection against giardiasis. Likewise, lack of formal education is associated with *P*. *falciparum* malaria among pregnant women in northern Ghana. The use of ITN and mosquito repellents reduce the risk of for *P*. *falciparum* malaria.

This study thus provides guidance to health authorities regarding some associated factors to prevent malaria and intestinal parasite infections among pregnant women in northern Ghana. The high prevalence of the parasites underscores the need to up-scale public health education to control parasitic infections among pregnant women in the study area. Public health education of pregnant women on personal and environmental hygiene, protection from mosquito bites through the use of ITN and mosquito repellents, and other practices that predispose to parasitic infection would be a relatively inexpensive mode to reduce the prevalence of the infection. Owing to the limited availability of health care facilities in the study region, we recommend regular screening and surveillance, and treatment of parasitic infections among pregnant women. This will not only improve the health, wellbeing and quality of life of the pregnant women but also help abate the plethora of complications on the unborn baby as a result of maternal parasitic infections.

## Supporting information

S1 Data (XLSX)
